# Three-Dimensional Finger Vein Recognition: A Novel Mirror-Based Imaging Device

**DOI:** 10.3390/jimaging8050148

**Published:** 2022-05-23

**Authors:** Christof Kauba, Martin Drahanský, Marie Nováková, Andreas Uhl, Štěpán Rydlo

**Affiliations:** 1 Department of Computer Sciences, University of Salzburg, Jakob-Haringer-Str. 2, 5020 Salzburg, Austria; uhl@cosy.sbg.ac.at; 2 Department of Intelligent Systems, Faculty of Information Technology, Brno University of Technology, Božetěchova 2, 612 00 Brno, Czech Republic; drahan@fit.vutbr.cz (M.D.); irydlo@fit.vut.cz (Š.R.); 3 Department of Physiology, Faculty of Medicine, Masaryk University, Kamenice 5, 625 00 Brno, Czech Republic; majka@med.muni.cz

**Keywords:** finger vein recognition, 3D vascular pattern recognition, multi-perspective finger vein capturing device, mirror-based capturing device, state-of-the-art

## Abstract

Finger vein recognition has evolved into a major biometric trait in recent years. Despite various improvements in recognition accuracy and usability, finger vein recognition is still far from being perfect as it suffers from low-contrast images and other imaging artefacts. Three-dimensional or multi-perspective finger vein recognition technology provides a way to tackle some of the current problems, especially finger misplacement and rotations. In this work we present a novel multi-perspective finger vein capturing device that is based on mirrors, in contrast to most of the existing devices, which are usually based on multiple cameras. This new device only uses a single camera, a single illumination module and several mirrors to capture the finger at different rotational angles. To derive the need for this new device, we at first summarise the state of the art in multi-perspective finger vein recognition and identify the potential problems and shortcomings of the current devices.

## 1. Introduction

Biometric recognition technology is a well-established way to provide secure access and to verify the identity of a person. The most commonly employed biometric recognition devices are based on fingerprint, face and iris recognition. Recently, other biometric traits, such as the vascular pattern of the human body, especially inside the fingers, also denoted as finger vein recognition, have become more popular.

In comparison to fingerprint recognition, finger vein recognition suffers from limitations due to the imaging method (mainly based on near-infrared illumination), especially low-contrast images due to light scattering and reflection. Several improvements have been made in recent years, especially in terms of usability and recognition accuracy, by utilising recent deep-learning-based techniques [1]. However, there is still a lot of room for improvements, especially if it comes to touch-less acquisition of the finger vein samples. Apart from the difficulties in the sample acquisition process, there are currently many more open questions, e.g., the influence of certain medical conditions (decreased blood oxygenation, e.g., due to anaemia or various cardiovascular and pulmonary disorders changing tissue perfusion) on the accuracy of finger vein recognition and also the influence of different environmental conditions (e.g., temperature, ambient illumination and humidity) on a finger vein recognition system. This work is not on introducing finger vein recognition and its advantages but on a specific sub-topic in finger vein recognition: 3D or multi-perspective finger vein acquisition. For more information about finger vein recognition in general, the interested reader is referred, e.g., to [1].

One main problem for many finger vein acquisition devices is misplacement/rotations in general, especially longitudinal finger rotation. All the classical well-established finger vein recognition algorithms and even more recent key-point-based and CNN-based ones are very sensitive to this kind of finger misplacement [2].

A way to tackle the problems of finger misplacement and rotation is to resort to a 3D or multi-perspective finger vein acquisition device. Such a device usually consists of several cameras and illumination units and captures the finger vein samples from several different rotational angles around the finger’s longitudinal axis, and in combination with a suitable reconstruction algorithm, a full 3D representation of the finger vein pattern can be obtained. A multi-perspective finger vein acquisition device achieves higher recognition performance compared to single-view ones and is robust against simple paper-printout presentation attacks [3].

A drawback of a multi-perspective acquisition device in contrast to the traditional ones, based on a single camera and illumination unit, is the increased cost and complexity of the capturing device due to the higher number of cameras and illumination units. Depending on the application scenario, one has to find a compromise between recognition accuracy, usability and complexity/cost of the acquisition device. The more user friendly the acquisition is (contactless, on the move), the more finger misplacement will occur. In such cases, the added cost and complexity of a multi-perspective acquisition device can be justified in order to achieve a good recognition accuracy.

The main purpose of this work is to present a new 3D finger vein acquisition device, which in contrast to the existing devices, is based on several mirrors in combination with only a single camera and illumination module. This helps to reduce the complexity and costs of the acquisition device. To the best of our knowledge, a multi-perspective finger vein acquisition device based on mirrors has not been proposed so far. After providing a short general introduction to finger vein recognition including a medical background on the vascular patterns within the human hands, the first part of this work is devoted to a summary of the current state of the art in 3D and multi-perspective finger vein recognition devices. Potential problems with the current devices will be identified and summarised in order to derive the need and the design for our novel capturing device, which should help to overcome some of the highlighted problems and shortcomings. In contrast to finger vein recognition and vascular pattern recognition in general, 3D or multi-perspective vascular pattern recognition is still quite new, and only a few research groups are working on this topic.

The rest of this work is organised as follows. Section 1 provides a short introduction and the medical background on finger vein recognition, followed by a summary of the current state of the art in 3D and multi-perspective finger vein acquisition including the potential problems and limitations. Section 2 presents our novel multi-perspective finger vein acquisition device and the current state of our evaluations. Finally, Section 3 concludes this paper and gives an outlook on future work.

## 2. State of the Art in Finger Vein Recognition

This section provides an overview of the state of the art in 3D and multi-perspective finger vein recognition. At first, the medical background on vascular pattern recognition and its properties is outlined, followed by a short description of the usual acquisition device design. The second part is then devoted to related works on 3D and multi-perspective finger vein recognition as well as the limitations and shortcomings with the current capturing devices.

### Medical Background on Vascular Pattern Recognition

The cardiovascular system plays an important role in numerous physiological functions in humans. It ensures transportation of substrates, energy, and signals throughout the body, thus helping to keep it in homeostasis. The heart, as a pump on one side, and the vessels, as the closed system of conduits on the other side, are a unique system, the functions of which are regulated by numerous nervous as well as humoral feedbacks. The heart consists of two pumps connected in series: the right ventricle drives the blood into the pulmonary (or so-called small) circulation, and the left ventricle—into the systemic (or so-called big) circulation.

The main role of the vessels is to guarantee proper perfusion, or in other words to ensure sufficient blood flow in the peripheral tissues. The term vessels comprises arteries of various types (e.g., elastic, muscular), the capillaries (forming so-called microcirculation) and the veins. These parts of circulation differ as far as the diameter, structure and thickness of the wall and the pressure in the lumen are concerned.

The diameter of the vessels decreases from 25–15 mm in the aorta to 8–5 μm in the capillaries and then again increases up to 30 mm in the v. cava. The structure of the wall reflects the key role(s) of the respective part of circulation. In the case of arteries, it is maintenance of the blood pressure gradient and consequently continuous blood flow during the whole cardiac cycle. Therefore, the elastic fibers dominate in the wall of both elastic and muscular arteries (with increasing muscular component in the latter) and the wall thickness varies from 2 mm in the aorta to 1 mm in smaller arteries. The role of capillaries lies in the exchange of substrates and blood gases between the blood and interstitial fluid. Thus, their wall is free of elastic or muscle fibers, and loose connections among the endothelial cells lining the inner surface of the capillaries enable lively exchange of various molecules. The thickness of the capillary wall varies around 0.5 μm. The veins are responsible for transportation of the blood back to the heart, and their wall thickness fluctuates between 1 μm in the venules to 1.5 mm in the v. cava. As far as the blood pressure is concerned, it can be simplified that it gradually decreases from the arteries (with the highest values during the systole in the aorta—approx. 120 mmHg) to the veins (with the lowest values during the diastole in the v. cava—5 mmHg).

Specific types of arteries—arterioles—are equipped with smooth-muscle sphincters, which enable them to actively change (e.g., after nervous stimulus) their diameter. This skill is a key condition for redistribution of the blood among different parts of circulation and for active blood flow change in certain physiological situations.

Blood flow in the vessels is kept by pressure gradient in the circulation, which naturally decreases from the root of the aorta to the v. cava as mentioned above. In cases where the blood pressure is kept constant, the blood flow depends on the vessel diameter: in the case of vessel narrowing (so-called vasoconstriction), the blood flow decreases, and in case of vessel widening (so-called vasodilation) the blood flow increases.

Blood flow changes reflect various physiological situations. Very illustrative is the situation in skeletal muscles during physical exercise: perfusion increases, and this so-called work hyperaemia also continues during the recovery period (time of this hyperperfusion depends on the duration of exercise and the workload). The blood flow may increase as much as 10–15× as compared to values before exercise, and it also reflects—in addition to the abovementioned—the type of exercise (dynamic vs. static). Increased blood volume in the vessels of skeletal musculature is provided by redistribution of blood from the gastrointestinal, reproductive, and excretory systems due to massive vasoconstriction in these circulations. It is interesting that massive vasodilation in skeletal muscles is caused by local chemical and thermal changes arising from the exercise (so-called metabolic vasodilation), not by whole-body regulatory circuits.

Another example of local blood flow increase is hyperaemia in the gastrointestinal tract during digestion and absorption. Very interesting—due to their ambiguity—are blood flow changes in skin circulation, which are part of the thermoregulatory response of the human body. In high ambient temperature or in cases of high internal production of heat (e.g., during physical exercise), blood flow increases; however, as a response to low ambient temperature, decreased blood flow in skin is observed, resulting from massive vasoconstriction on skin circulation.

Aging represents a complex of physiological changes. Its onset and degree evince very high interindividual variability. Aging changes both structure and, consequently, function(s) of most systems, including the vessels. One of major signs of aging is changes in the compliance of the arteries, based on the loss of elastic fibres in the wall and their replacement by collagen fibres. Additionally, crosslinking among collagen fibres in the arterial wall is increasing and progressive and diffuse fibrotic changes are observed. Thus, the arterial walls become less compliant, e.g., less distensible; in other words, they are stiffer than younger arteries of the same person. Old arteries can accommodate a smaller volume of blood at a given blood pressure than young arteries. This situation can be viewed from other side: to accommodate the same blood volume as the young artery, the blood pressure in the older artery is higher. Blood flow through a physiologically aged vessel is often compromised by one of the most frequent diseases of cardiovascular system—atherosclerosis.

Changes in blood flow can be also part of other pathological situations. The list of such disorders contains various metabolic, endocrine, and cardiovascular diseases or vessel malformations and changes caused by injuries. During changed blood flow, the detection of vessels may be partially or completely compromised.

## 3. Blood Vessels in the Hand

**Figure 1:**
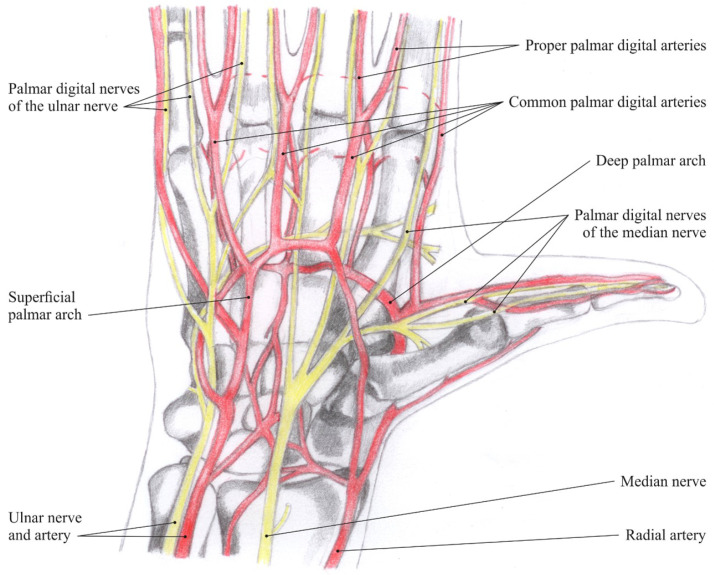
Nerves and arteries of the hand [4].

Blood supply in the hand area is ensured by two arteries—the radial and ulnar arteries. The former supplies blood mostly to the thumb and lateral side of the index and the latter to the remaining parts of the hand; both these arteries anastomose and form two carpal networks (palmar and dorsal) and two palmar arches (superficial and deep). These vessels then supply blood to the muscles, joints and digits of the hand (Figure 1: Nerves and arteries of the hand).

**Figure 2:**
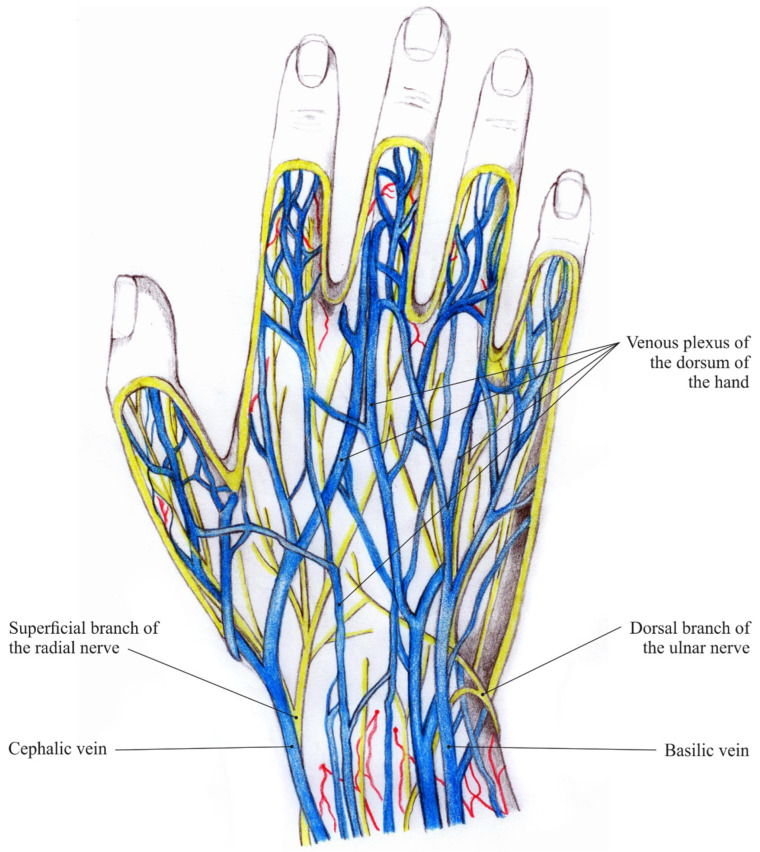
Veins and nerves of the hand dorsum [4].

Deep and superficial veins in the hand are interrelated via communicating veins. The deep veins convoy the hand arteries and are named similarly to them. The superficial veins form the venous plexuses in the subcutaneous tissue, which then constitute the cephalic vein and the basilic vein (Figure 2: Veins and nerves of the hand dorsum). Striking visible symptom of aging hands are ectatic veins. They become even more prominent when, due to physiological aging, dermal thickness decreases and subcutaneous tissue is lost [5].

### 3.1. Traditional Finger Vein Capturing Devices

The vascular patterns inside the fingers need to be made visible and captured by a suitable biometric capturing device, usually denoted as a finger vein scanner. These devices harvest the fact that the de-oxinated haemoglobin in the blood flowing through the blood vessels has a higher light absorption coefficient within the near-infrared spectrum than the surrounding tissue.

Hence, the common set-up of a finger vein capturing device includes a near-infrared sensitive camera and a near-infrared illuminator with a light emission peak wavelength between 700 nm and 950 nm. Most finger vein capturing devices capture the palmar (bottom) side of the finger using the light transmission principle, where the camera and the illumination unit are placed on opposite sides of the finger. Some devices are capturing the dorsal (top) side of the finger, while the other views around the finger’s longitudinal axis are rarely used, especially in commercial devices. Recently, the trend has been moving towards fully contactless devices, which do not have any physical finger positioning aids and improve users’ acceptance.

Although there is no reason to believe that there is a real anatomical difference in the blood vessels among the fingers, usually the index, middle and ring fingers are used in finger vein recognition as those are the most convenient to provide to the capturing device for the users and are longer compared to the thumb and little finger. According to [6], there is no best finger, as the best-performing finger depends on the particular recognition algorithm and the dataset. Hence, each data subject has a best-performing finger in certain scenarios and there is no general best-performing finger for all users.

#### Background on the Type of Illumination

**Figure 3:**
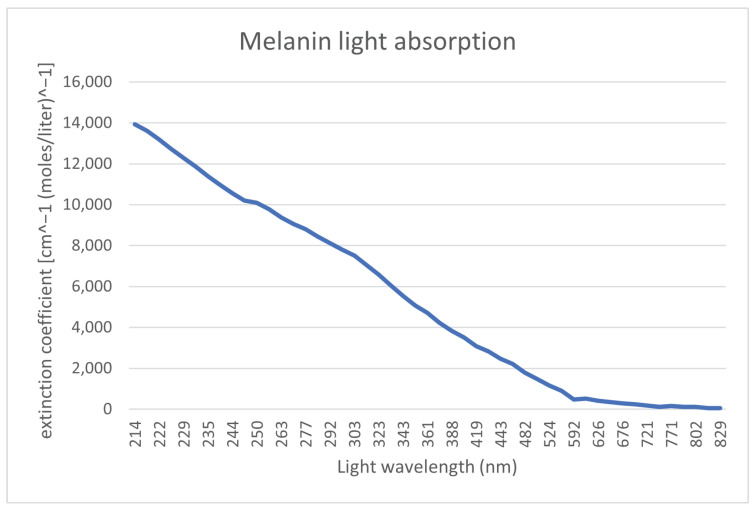
Melanin light absorption [7].

As mentioned in the previous subsection, finger vein capturing devices use light sources in the NIR spectrum. To justify this choice, we have a look at each layer of the skin and the location of the bloodstream, in particular at the chemicals and substances that are most represented in a given skin layer. For these chemicals, our focus is on their light absorption at a particular wavelength, which can be defined as a molar extension coefficient. The bloodstream is located in the second skin layer (dermis), which is underneath the first layer of the skin (epidermis). To render the bloodstream visible, a light spectrum, which penetrates through the first layer of the skin and reaches the bloodstream, is necessary. This first layer of the skin (epidermis) contains ordinary melanin. Figure 3 shows the absorption levels of melanin. It can be seen that the absorption is close to 0 starting from 800 nm light wavelength. Hence, to penetrate the first layer of the skin, a light source with a wavelength around 800 nm or higher should be used.

**Figure 4:**
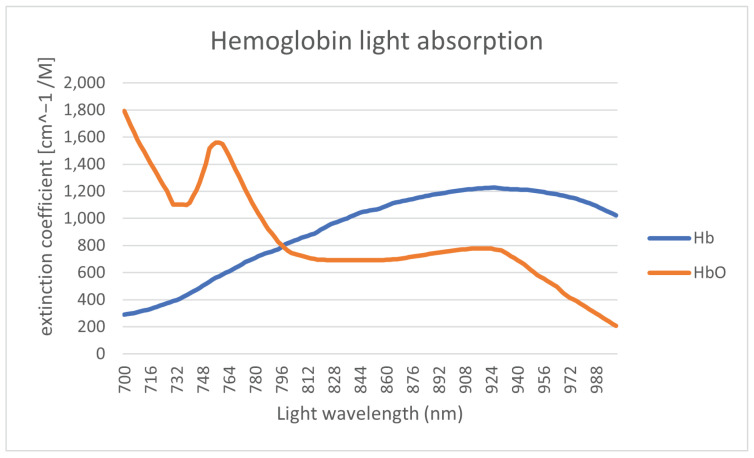
Oxygenated and de-oxygenated hemoglobin light absorption [8].

The primary substance of the bloodstream is hemoglobin, which is contained in the red blood cells. There is a different molar extension coefficient for oxygenatedand de-oxygenated hemoglobin. The light absorption of the hemoglobin is shown in Figure 4.

The range of the wavelength of the light spectrum shown in Figure 4 is restricted to the range of 700–1000 nm due to melanin limitation. The differences in light absorption levels between oxygenated (Hb) and deoxygenated (HbO_2_) hemoglobin are then utilized to define the saturation. The saturation level registered by medical equipment is defined by the ratio between this two variants of hemoglobin. For high-quality recognition, ideally a light wavelength close to the intersection of these two curves should be used. The next limitation of the illumination light wavelength (in terms of absorption) is substances such as fat, where the absorption is significantly increasing for wavelengths above 900 nm [9]. Fat is mainly contained in the next layer tissue layer (third layer) underneath the one containing the bloodstream.

### 3.2. Three-Dimensional and Multi-Perspective Vascular Imaging

Apart from the usual single-view finger vein capturing devices, several researchers have developed different 3D or multi-perspective finger vein capturing devices. These works are summarised in the following, structured according to their operation principle, starting with rotating devices, followed by multi-camera devices and finally, related works that are dedicated either to other principles of capturing devices or to processing the multi-perspective finger vein data.

#### 3.2.1. Rotation-Based Multi-Perspective Finger Vein Capturing Devices

Qi et al. [10] proposed a 3D hand-vein-capturing system based on a rotating platform and a fixed near-infrared camera, which is located above the hand. The hand is put on a handle (which is mounted on the rotating platform) with an integrated illuminator based on the light transmission principle. The plate rotates around the z-axis. However, the degree of rotation is limited due to the limited movement of the hand in this position. A 3D point cloud is generated from the samples captured at different rotation angles. The 3D point-cloud-based templates are compared using a kernel correlation technique. They conducted some recognition performance evaluation using a small dataset (160 samples) captured with their proposed device, but no comparison with other hand-vein recognition algorithms on their acquired hand-vein samples. The authors claim that their approach should help to overcome hand registration and posture change problems present in hand-vein recognition if only 2D vein patterns/images are available.

**Figure 5:**
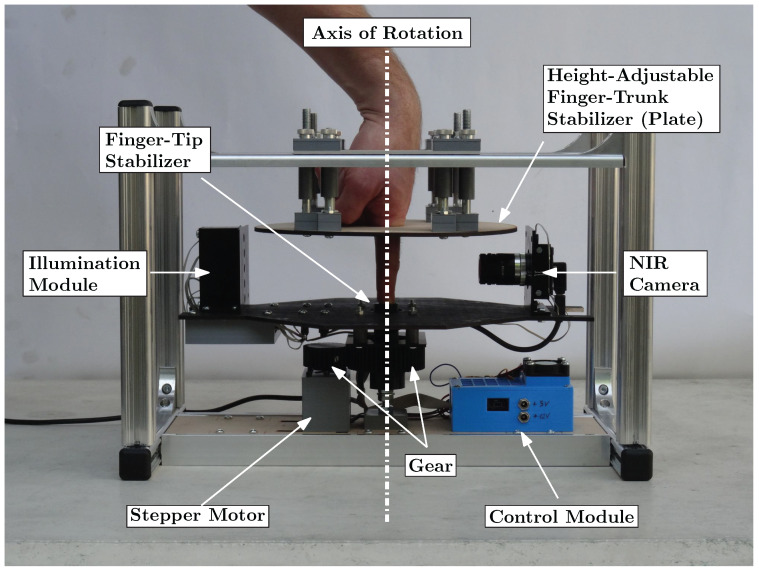
Self-designed multi-perspective finger vein capture device (image originally published in [2]).

In 2018, Prommegger et al. [2] constructed a custom, rotating 3D finger-vein-capturing device. This capturing device acquires finger vein images in different projection angles by rotating a near-infrared camera and a illumination unit around the finger. The principle of the device is shown in Figure 5. The finger is positioned at the axis of rotation, while the camera and the illumination module are placed on the opposite sides, rotating around the finger. The scanner is based on the light transmission principle and uses an industrial near-infrared enhanced camera and five near-infrared laser modules (808 nm) as light source. The acquisition process is semi-automated. After the finger has been inserted into the device, the operator has to initiate the capturing process. The illumination for the finger is then set automatically in order to achieve an optimal image contrast with the help of a contrast measure. Afterwards, the acquisition is started at one video frame per degree of rotation; hence, a full capture includes 360 images, one image per degree of rotation. All perspectives are captured in one run using the same illumination conditions to ensure the comparability of the different captures at different projection angles.

**Figure 6:**
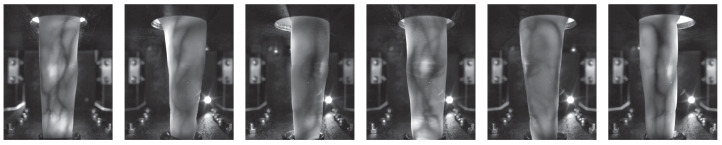
Multi-perspective finger vein dataset example images, from left to right: 0°, 60°, 120°, 180°, 240°, 300° (image originally published in [2]).

With the help of this custom rotational scanner, they established the first available multi-perspective finger vein dataset, consisting of 252 unique fingers from 63 volunteers, each finger captured 5 times at 360 perspectives (the 0° and 360° perspective are the same); hence, it contains 252×361×5=454.860 images in total. Each perspective is stored as an 8-bit grayscale image with a resolution of 1024 × 1280 pixels. The finger is always placed in the centre of the image; hence, the sides can be cut, resulting in an image size of 652 × 1280 pixels. Figure 6 shows some example images of this dataset. For more details on the dataset and the capturing device, refer to our original work [2].

Based on this dataset, they evaluated the recognition performance of the single perspectives as well as a score level fusion based on 2, 3 and 4 perspectives using well-established vein-pattern-based recognition algorithms. Their results confirmed that the palmar perspective (0°) achieved the best performance, followed by the dorsal one (180°), while all other perspectives are inferior.

#### 3.2.2. Multi-Camera-Based Multi-Perspective Finger-Vein-Capturing Devices

In 2013, Zhang et al. [11] proposed one of the first binocular stereoscopic vision devices to capture hand vein images and the shape of the finger knuckles. The recognition is based on a 3D point cloud matching of hand-veins and knuckle shape. Their capture device set-up consisted of two cameras, placed in a relative position of about 45° next to each other, each one equipped with an NIR-pass filter. There is only a single light transmission illuminator placed underneath the palm of the hand. The 3D point clouds are generated by extracting information from the edges of the hand veins and knuckle shapes. The generated 3D point clouds are then compared utilising a kernel correlation method, especially designed for unstructured 3D point clouds. The authors claim that their proposed method is faster and more accurate compared to 2D vein recognition schemes, yet they did not compare to other hand-vein recognition schemes.

Lu et al. [12] proposed a finger vein recognition system using two cameras. These cameras are placed separated by a rotation angle of 60° next to each other, where each camera is located 30° apart from the palmar view. They applied feature- as well as score-level fusion (based on the min and max rule) using the samples captured the two views simultaneously by the two cameras and were able to improve the recognition performance over samples captured from only one view.

Sebastian Bunda [13] proposed a method to reconstruct 3D point cloud data from the finger vascular pattern. His work is based on a a multi-camera finger-vein-capturing device constructed by the University of Twente. The University of Twente finger-vein-capturing device is also described in [14]. The device is based on the light transmission principle and captures the finger from the palmar side. It consists of three independent cameras located at the bottom of the device and separated by a rotation angle of about 20°. On the top of the device, there are three near-infrared LED illuminators, also separated by a rotation angle of 20°. The cameras have a field of view of 120° in order to capture the whole finger from a close distance and are equipped with NIR pass filters to block the ambient light. The same as the device proposed by [15], this device also requires a camera calibration and stereo rectification prior to any further processing of the acquired samples. After those steps, the finger vein lines are extracted (cropping the image, histogram equalisation, Gaussian filtering and then using the repeated line tracking [16] algorithm) from the pair of stereo images. According to the basic stereo vision principles, finger vein lines in the first image should be matched with the corresponding vein lines in the second image. Hence, a standard stereo correspondence algorithm is used to generate the disparity maps and derive the 3D point cloud data from those disparity maps. Note that so far only two pairs of cameras (cam1 and cam2 plus cam2 and cam3) are used to generate the 3D point clouds. Bunda evaluated his three-dimensional model visually and using a flat test object and a round test object. Tests with real fingers showed that the presented method is a good first attempt at creating 3D models of the finger vein patterns. On the other hand, he notes that there is still room for improvement, especially in terms of speed and accuracy. He did not conduct any recognition performance evaluations and there are no hints towards a full dataset captured with their scanner so far.

In order to address the limited vein information and the sensitivity to positional changes of the finger, another finger-vein-capturing device with an accompanying finger vein recognition algorithm in 3D space was proposed by Kang et al. [17]. The authors claim that their capturing device is able to collect a full view of the vein pattern information from the whole finger and that their novel 3D reconstruction method is able to build the full-view 3D finger vein image and the corresponding 3D finger vein feature extraction from the samples acquired by their capturing device. Their capturing device consists of three cameras (placed at an rotation angle of 0°, 120° and 240°) and three corresponding illumination units (light transmission), which are placed on the opposite side of the camera. The illuminators use NIR LEDs with a wavelength of 850 nm and the cameras are equipped with an 850 nm NIR band-pass filter. The authors point out that using a standard feature-based stereo matching technique for finger vein recognition is difficult as it is hard to find a sufficient number of matched points between two images to obtain all of the deep-vein information, while area-based stereo-matching techniques have difficulties in extracting the vein lines robustly. Hence, they suggest a novel 3D representation as well as a feature extraction and matching strategy based on a lightweight convolutional neural network (CNN). At first, similar to other approaches, their set-up requires a camera calibration and stereo rectification. Afterwards, they generate elliptical approximations of the finger’s cross section at each horizontal point in the 2D images based on the detected finger outline in the three captured 2D images. Using those ellipses and the three 2D images, they can then establish a 3D coordinate system and generate a 3D finger model of the whole finger. Once this is completed, they can map the texture information of the 2D finger vein images onto the 3D finger model. Afterwards, a 3D finger normalization is applied, which eliminates variations caused by Y-shift or Z-shift movements and pitch or yaw movements, with only the variations caused by rotation along the X-axis (roll movement) and X-shift movement remaining. These problems are addressed in the subsequent feature extraction and comparison step, which utilizes two different CNNs to extract the vein texture information and the 3D finger geometry from the 3D representation. Those features are then compared using a CNN to establish the final comparison score after fusing the two separate scores. The authors also established a 3D finger vein database containing 8526 samples. Their experimental results demonstrate the potential of their proposed system. They also did comparisons with classical finger vein recognition approaches and three CNN-based ones and showed that their approach is superior in terms of recognition accuracy. Despite the promising results, the authors consider their work as a “preliminary exploration of a 3D finger vein verification system” and point out that substantial improvements still need to be made in order to improve the verification accuracy and to reduce the time consumption. An improvement in image quality is essential, which requires an improvement in their capturing device hardware, including the camera performance as well as the light path design and illumination control. Furthermore, the 3D reconstruction and texture mapping procedures introduce some errors, e.g., related to non-rigid transform, uneven exposure and shift transforms, which need to be addressed.

In [18], Prommegger et al. derived a capturing device design that achieves an optimal trade-off between cost (number of cameras) and recognition performance. Based on the single-view scores of [19], they carried out several score-level and feature-level fusion experiments involving different fusion strategies in order to determine the best-performing set of views and feature extraction methods. The results showed that by fusing two independent views, in particular, the palmar and dorsal view, a significant performance gain was achieved. By adding a second feature extraction method to the two views, a further considerable performance improvement is possible. Taking the considerations about capturing time and capturing device costs into account, the preferred option is to design a two-perspective capture device capturing the palmar and dorsal view, applying a two-algorithm fusion including Maximum Curvature and SIFT. The second feature extraction method can be included without involving additional hardware costs just by extending the recognition tool chain. The drawback of this capturing device design is that a real 3D reconstruction of the vein structures will not be possible, so if that is taken into account, the capturing device should consist of at least three different cameras and three illumination units as, e.g., proposed by Veldhuis et al. [14].

Durak et al. [20] designed and constructed a 3D finger vein scanner for implementing an authentication mechanism for secure strong access control on desktop computers based on biometry. Their system implements privacy-by-design by separating the biometric data from the identity information. Their 3D finger-vein-capturing device was constructed in cooperation with Global ID, IDIAP, HES-SO Valais–Wallis, and EPFL and captures the finger from three different perspectives (the palmar perspective and two further perspectives that are separated by an rotational angle of 60° to the palmar one). It uses three cameras but only one illumination unit on top of the device (opposite to the palmar camera). No further details about their 3D finger-vein-capturing device are available as the rest of the paper is about the implementation of the security protocols. The device seems to be at least similar to the one constructed by the University of Twente [13,14].

The LFMB-3DFB is a large-scale finger multi-biometric database for 3D finger biometrics established by Yang et al. [21]. They designed and constructed a multi-view, multi-spectral 3D finger imaging system that is capable of simultaneously capturing finger veins, finger texture, finger shape and fingerprint information. Their capturing device consists of six camera/illumination modules, which are located in a rotational distance of 60° to each other around the longitudinal axis of the finger (with the first camera being placed at the palmar view). The illumination pars consist of blue and near-infrared light sources, to be able to capture both the vein and the skin texture information. With the help of their proposed capturing device, they established LFMB-3DFB by collecting samples of 695 fingers from 174 volunteers. They also propose a 3D finger reconstruction algorithm which is able to include both finger veins as well as skin textures. As usual for multi-camera systems, at first an internal and external camera parameter calibration has to be performed. Afterwards they employ a K-Means, Canny edge detection and guided-filter-based finger contour segmentation, which is refined using a CNN in a second stage. The next step is a finger visual hull reconstruction based on the Space Carving algorithm, which is a simple and classical 3D reconstruction algorithm. In the following, a template-based mesh deform algorithm is applied in order to remove sharp edges and artefacts introduced by the visual hull reconstruction. The template used to represent the finger is a cylindrical mesh. Finally, a texture mapping stage is performed, where the skin and vein textures are mapped to the vertices of the 3D finger mesh. The authors performed several recognition performance experiments based on the single-perspective samples, the reconstructed 3D finger vein models, on 2D multi-perspective samples and a fusing (score- as well as feature-level fusion) of the former ones using several CNN-based feature extraction and comparison approaches. Their results suggest that the best recognition performance can be achieved by using the multi-perspective samples in combination with feature-level fusion, but the performance of the 3D end-to-end recognition using their second CNN approach is almost as good as the multi-perspective one. They account the inferior performance of their 3D end-to-end approach to problems with the accuracy of their 3D finger reconstruction algorithm as well as problems with the 3D end-to-end recognition CNN, which they want to improve.

Quite recently, in December 2021, Momo et al. patented a “Method and Device for Biometric Vascular Recognition and/or Identification” [22]. From the title of the patent, it is not obvious that this device performs 3D or multi-perspective finger vein recognition. The finger vein capturing device design in the patent is similar to the one presented by [20] as it uses three cameras placed at the palmar view and separated by a rotational angle of ±45°. It also uses only one illumination module, which is placed opposite to the palmar view camera, on top of the device. The patent claims that the device includes anti-spoofing measures and that the recognition is performed based on 3D finger vein templates where the vein information is extrapolated from a disparity map or by triangulation using two images provided by the cameras. For recognition, the patent only suggests that this can be performed based on “(deformable or scale-invariant) template matching, veins feature matching and/or by veins interest point detection and cross-verification”, so it does not give any further details about the feature extraction/comparison process.

The most recent publication on 3D finger vein recognition is the work completed by Xu et al. [23], published on the 4th of February, 2022. The authors propose a 3D finger vein verification system that extracts axial rotation invariant features to address the problem of changes in the finger posture, especially in contactless operation. This work is based on the authors’ previous work [17], i.e., it uses the same capturing device and also utilizes the dataset presented in [17] for performance evaluations. In contrast to their previous work, which had problems with the accuracy of the 3D finger vein model, here, the authors propose a silhouette-based 3D finger vein reconstruction optimization model to obtain a 3D finger point cloud representation with finger vein texture. This method is based on the assumption that the cross-section of finger approximates ellipses and employs finger contours and epipolar constraints to reconstruct the 3D finger vein model. The final 3D model is obtained by stacking up ellipses from all cross-sections. Finally, the vein texture is mapped to the 3D point cloud by establishing the correspondences between 3D point cloud model and the input 2D finger vein images. The next step is to input the 3D point clouds to a novel end-to-end CNN network architecture called 3D Finger Vein and Shape Network (3DFVSNet). The network transforms the initial rotation problem into a permutation problem and finally transforms this to invariance (against rotations) by applying global pooling. These rotation-invariant templates are then compared to achieve the final matching result. The authors also extended their initial dataset (SCUT-3DFV-V1 [17]) to the new SCUT LFMB-3DPVFV dataset, which includes samples of 702 distinct fingers and is available on request. The authors claim that this is so far the largest publicly available 3D finger vein dataset and is three times larger than their initial one. They performed several experiments on both of their datasets (SCUT-3DFV-V1 and SCUT LFMB-3DPVFV) and showed that their proposed 3DFVSNet outperforms other 3D point-cloud-based network approaches (e.g., PointNet). They also compared their results with well-established vein recognition schemes and showed that their 3DFVSNet performs better on all tested benchmarks on the SCUT-3DFV-V1 and LFMB-3DPVFV datasets.

#### 3.2.3. Other Multi-Perspective Finger-Vein-Capturing Devices and Data Processing

A completely different approach to 3D finger vein authentication was presented by Zhan et al. [24]. Their approach is not based on the widely employed near-infrared light transmission principle but on photoacoustic tomography and is an improvement over their previous work on an photoacoustic palm vessel sensor [25]. They presented a compact photoacoustic tomography setup and a novel recognition algorithm. Compared to optical imaging technologies, the photoacoustic tomography (PAT) is able to detect vascular structures that are deeper inside the tissue with a high spatial resolution because the acoustic wave scattering effect is three orders of magnitude less than the optical scattering in the tissue. Optical imaging suffers from light diffusion, while ultrasound imaging suffers from low sensitivity to blood vessels. PAT is able to output images with a higher signal-to-noise ratio (SNR) because non-absorbing tissue components will not generate any PA signals. The authors propose a finger-vein-capturing device that is able to cover four fingers (index, middle, ring and pinky) at a time and allows the data subjects to place their fingers directly on top of the imaging window to enhance the usability. The data subject’s fingers are placed on top of a water tank. The optical fiber output and ultrasound transducer are located inside the water tank. The ultrasound transducer has 2.25 MHz centre frequency and a 8.6 cm lateral coverage to be able to cover all four fingers and the device uses a laser with a wavelength of 1064 nm as light source. Both the light delivery and the acoustic detection path are combined using a dichroic cold mirror, which also reflects the photoacoustic signals generated by the finger vessels. To scan the whole finger, the laser is moved along the finger during a single capture. Using this PAT-based finger vein capturing device, the authors collected samples of 36 subject to establish a test data-set. The software part of their proposed system includes the reconstruction of the 3D image from the acquired 2D images at each laser pulse by stacking all the 2D data based on their position and finally depth-encoding and maximum-intensity-projecting the data. Afterwards, a vessel structure enhancement is conducted by at first de-noising and smoothing the original reconstructed image using Gaussian and bilateral filters. Then, a 3D vessel pattern segmentation technique, which is essentially a binarisation method, including a nearest-neighbor search and vascular structure fine-tuning module (skeleton) is applied. The finger vein templates are then binary, 3D finger vein representations. For comparison, a SURF key-point-based approach is employed. Note that while the vessel structure is essentially 3D information, the SURF features are intended for the 2D space. The authors conducted some verification experiments to determine the recognition performance of their approach and achieved accuracies of more than 99% or an EER of 0% and 0.13% for the right and left index finger, correspondingly. However, they did not compare their results to the well-established finger vein recognition schemes. A drawback of their capturing device design is the production costs due to the photoacoustic tomography setup and the parts that are needed to establish this setup.

Liu et al. [26] presented an approach for the efficient representation of 3D finger vein data. Instead of the typical point cloud representation, they propose a so-called liana model. At first, they store the finger vein data in a binary tree, and then they propose some restrictions and techniques on the 3D finger vein data, which are caliber uniformity (represent each vein line with the same thickness), bine classification (veins are classified gradually from root to terminal, palmar to dorsal surface, and counter-clockwise), node partition (usually two smaller veins intersect and merge into a thicker one, which leads to a 3-connectable node, all other nodes should be partitioned into 3-connectable ones) and spread restriction (vein loops can occur if the veins are spread around the finger skeleton, which are not allowed in a binary tree representation; hence, spread restriction removes the loop structures without discarding any vein information) in order to simplify the representation. The authors did not capture any 3D finger vein data, nor did they conduct any empirical (recognition performance) evaluation of their proposed 3D finger vein representation.

Another point-cloud-based 3D finger vein recognition technique was proposed by Ma et al. [15]. They strive to augment the finger vein samples by solving the problem of the lack of depth information in 2D finger vein images. They constructed a finger-vein-capturing device using two cameras that are positioned along the finger (not the longitudinal axis) in a binocular set-up, which is able to perceive the depth information from the dorsal view of the finger, but no full 360° 3D vein information is available as only the dorsal perspective is captured. After applying a camera calibration and stereo rectification for their proposed capturing device set-up, they extract the vein contours and the edges of the finger as features. Based on those features and the pair of stereo images, a 3D point cloud is reconstructed. Comparison of two samples is performed by utilizing the iterative closest point (ICP) algorithm to compare the two 3D point-cloud-based templates. The authors claim that due to the more discriminative features in their 3D point cloud finger vein representation, the recognition performance (matching accuracy) is enhanced. They performed neither any recognition performance evaluations, nor a comparison with other finger vein recognition schemes.

In [19], based on the multi-perspective finger vein data acquired in [2], the authors evaluated the effect of longitudinal finger rotation, which is present in most of the commercial off-the-shelf and custom-built finger-vein-capturing devices. This effect describes a rotation of the finger around its longitudinal axis between the capturing of the reference and the probe template. They showed that most of the well-established vein-pattern-based recognition algorithms are only able to handle longitudinal finger rotation to a small extent (±5°) before their performance degrades noticeably. The key-point-based ones (SIFT and DTFPM [27]) are able to compensate a higher level of longitudinal finger rotation (±15°), but for longitudinal finger rotations of more than 30°, the recognition performance degradation renders the tested finger vein recognition schemes useless.

A natural extension to this work was to analyse the amount of longitudinal finger rotation present in available finger vein datasets (UTFVP [28], FV-USM [29], SDUMLA-HMT [30] and PLUSVein-FV3 [31]) in order to show that longitudinal finger rotation is present in current finger vein datasets. In [32], the authors proposed an approach to estimate the longitudinal finger rotation between two samples based on their rotation correction approach introduced in [33] and found that especially the FV-USM and the SDUMLA-HMT are subject to a great amount of longitudinal finger rotation. A more sophisticated approach to detect the longitudinal finger rotation was presented in [34] and is a CNN-based rotation detector able to estimate the rotational difference between vein images of the same finger without requiring any additional information. The experimental results showed that the rotation detector delivers accurate results in the range of particular interest (±30°), while for rotation angles >30°, the estimation error rises noticeably.

In [33], the authors suggest several approaches to detect and compensate the longitudinal finger rotation, e.g., using the known rotation angle, overusing a geometric shape analysis, elliptic pattern normalisation and just by using a fixed rotation angle (independent of the real rotation angle).

In [35], the authors propose a method to generate a reference finger vein template that makes the probe template during the authentication purpose longitudinal rotation invariant. Their method, called multi-perspective enrolment (MPE), acquires reference samples in different rotation angles and then applies the CPN method proposed in [33]. For authentication, only a single perspective is acquired and the extracted template is compared to all enrolment templates where the final comparison score is the maximum score of all the comparisons. The second technique for rotation invariant probe templates proposed in [35] also captures several reference samples from the finger in different rotation angles, which are then normalized using CPN, but then combines the different perspectives by stitching the templates to form a so called perspective cumulative finger vein template (PCT). This template can be thought of as a “rolled” finger vein template, which contains the vein information of all around the finger. During the authentication, the comparison score is calculated by sliding the probe template over the cumulative reference template. This work is extended in [36] by a method to reduce the number of different perspectives during enrolment of a longitudinal rotation invariant finger vein template, and thus a way to reduce the number of cameras and illumination units equipped in a finger vein capturing device. This new method is based on the good results we achieved with our multi-perspective enrolment (MPE) method in [35], as well as the insights of the optimal number of different perspectives gained in [18]. The new method is called Perspective Multiplication for Multi-Perspective Enrolment (PM-MPE) and essentially combines the MPE with the fixed angle correction method introduced in [33]. They showed that PM-MPE is able to increase the performance over the previously introduced MPE method. In [37], they extended the work on PM-MPE presented in [36] by applying it to other feature extraction schemes (was only evaluated using Maximum Curvature in [36]) and by introducing so-called perspective shifts to MPE as well as by adding more than two pseudo perspectives in between each of the cameras for PM-MPE. Their experimental results showed that the perspective shifts on their own are not able to significantly improve the performance, but if the PMx-MPE is employed (additional pseudo perspectives), then especially the well-established vein-pattern-based systems benefit most from the insertion of additional pseudo perspectives.

In [3], Prommegger et al. presented a novel multi-camera finger vein recognition system that captures the vein pattern from multiple perspectives during enrolment and recognition (as a further extension to our previously proposed MP-MPE approach). In contrast to existing multi-camera solutions using the same capturing device for enrolment and recognition, the proposed approach utilises different camera positioning configurations around the finger of the capturing device during enrolment and recognition in order to achieve an optimal trade-off between the recognition rates (minimising the rotational distance between the closest enrolment and recognition sample) and the number of cameras (perspectives involved) needed. This new approach is called Combined Multi-Perspective Enrolment and Recognition (MPER) and is designed to achieve fully rotation-invariant finger vein recognition. The same as the previous MP-MPE approach, the robustness against longitudinal finger rotation is achieved by acquiring multiple perspectives during enrolment and now also during recognition. For every recognition attempt, the acquired probe samples are compared to the corresponding enrolment samples, which leads to an increased number of comparisons in contrast to the MP-MPE approach. The proposed capturing devices for MPER were not actually built, but have been simulated using the PLUSVein-FR dataset [2], which allowed them to easily test a group of different sensor configurations in order to find the best-performing one. Their experimental results confirmed the rotation invariance of the proposed approach and showed that this can be achieved by using as few as three cameras in both devices. This method is solely based on well-established vein recognition schemes and a maximum rule score level fusion and thus, does not need any costly 3D reconstruction and matching methods like other proposed multi-camera recognition systems, e.g., [17], do. The computation time of our proposed MPER approach has comparable results with existing solutions and can be used in real-world applications. MPER enables both the enrolment and the recognition-capturing device to be equipped with three cameras. Most of the other proposed multi-perspective finger-vein-capturing devices use three cameras as well [13,17,22]. A comparison with those systems shows that the actual placement of the cameras is important as well. If the same methodology as that proposed for MPER is used for these proposed capturing devices [13,17,22], only the configuration of MPER-120°/3 achieves rotational invariance.

### 3.3. Problems and Shortcomings of the Current Capturing Devices

All available commercial off-the-shelf devices for vascular recognition (finger and hand vein ones) are based on one angle of view (usually the palmar view). This means that the recognition is based on a 2D image, leading to all the problems inherent with longitudinal finger rotation and finger misplacements. One way to overcome these limitations and to increase the recognition accuracy is to use 3D or multi-perspective capturing devices. These capturing device designs are summarised in the above-mentioned related works.

The goal of the capturing device design is to maintain a good usability of the system, especially keeping acquisition time low (which is a main drawback of the rotational capturing device designs) while achieving the highest possible accuracy, usually involving the best possible sample image quality. Another important aspect is the complexity of the capturing device, which is directly related to its production costs. For practical applications, a trade-off between performance (recognition accuracy), usability (user friendliness) and costs has to be found.

In terms of capturing device set-ups for 3D or multi-perspective vascular-pattern-capturing devices, there are three different kinds of devices: rotational devices, devices based on multiple cameras placed at different angles and devices based on non-optical sensing techniques, e.g., ultrasound. For each of these types, there are several examples in the literature, with each one of those having its disadvantages, which will be discussed in the following sections.

The rotating finger vein scanner presented in [2] is one example of the first group of devices where the camera rotates around the finger, while Qi et al. [10] presented a device with a fixed camera and a rotating platform, i.e., the hand rotates. The main disadvantage of the rotational devices is high capturing time, as the samples have to be acquired sequentially during the rotation. On the one hand, this is bad in terms of usability (users’ acceptance), but on the other hand it is prone to motion blur and involuntary finger movements during the acquisition, both significantly degrading the acquired samples’ quality and thus the recognition performance. Another problem is the complexity of the rotation mechanism, making the device more prone to mechanical failures, especially over time, and increasing the construction complexity. On the positive side, the parts costs are lower compared to the multi-camera devices, as one camera and one illumination unit are sufficient.

The most prominent group of devices are the multi-camera ones, which can have two [11,12,15], three [13,14,17,20,22,23], or even up to six [21] cameras and corresponding illumination modules. The obvious advantage of those devices is that a sample acquisition can be conducted in parallel with all the cameras (depending on the device’s configuration) and is thus much faster than for the rotational devices. The main drawback of those devices is the increased complexity and costs due to the multiple cameras and illumination modules, also requiring more complex control electronics to handle the image capturing and illumination control. The costs usually increase linearly with the number of cameras/illumination units as usually each camera has its own light source. Depending on the placement of the cameras and illumination modules, the sample acquisition might still need several sequential steps as the different illumination modules might interfere with each other. In particular, the systems based on two cameras require a stereo calibration before, and the stereo rectification process introduces additional distortions into the acquired samples, lowering the sample quality. Systems with only two cameras are also not able to capture the full 3D image around the finger. The number of perspectives, and thus, the recognition accuracy (or also the accuracy of the 3D reconstruction) can be increased by increasing the number of cameras, but the maximal number of cameras is limited by the available space within the device.

A representative of the last group of devices is, e.g., the device proposed by Zhan et al. [24], which is a successor of the previous device by Wang et al. [25] and based on photoacoustic tomography instead of near-infrared light. The main advantage of these devices is that photoacoustic tomography (PAT) is able to detect vascular structures that are deeper inside the tissue with a higher spatial resolution as compared to the near-infrared based ones. The main drawback of those devices are their high costs, which are in the range of EUR 10k and thus, 5–20 times higher than for the other two groups of devices.

## 4. Proposed 3D Vascular Pattern Capturing Device

The main idea behind the newly proposed capture device is to overcome some of the limitations/drawbacks of the existing devices, i.e., mainly reducing the costs while keeping the acquisition time low and the sample quality high. Our proposed device strives to achieve this by taking as much information as possible in one image using a single camera only, while the camera should still capture multiple perspectives around the longitudinal axis of the finger. Based on this idea, we need to derive the optimal set-up of the camera and light sources in order to minimise the costs and maximise the sample quality (i.e., minimising the distortions). So, first of all we need to find out the maximum possible angle between the light source and camera, within the visibility of finger veins. This information will provide us the optimal angle for capturing the finger veins with a single light source instead of placing multiple light sources, which will decrease the number of light sources in comparison to, e.g., the device from the University of Twente [13]. Afterwards, we need to find a way to maximise the number of views that can be captured with one fixed non-rotating camera, which can usually only capture one view. Our solution for capturing multiple views with one camera is to increase the field of view of this camera by using reflecting surfaces, e.g., mirrors. Mirrors have the advantage of being much cheaper as compared to employing multiple cameras, and by only having one non-moving camera in combination with the mirrors, the acquisition of all the perspectives around the finger can still be completed in a single shot acquisition. To minimise the optical distortions introduced by the mirrors, an optimal set-up in terms of mirror type and placement needs to be found, which is described in the following.

### 4.1. Test Device and Results

**Figure 7:**
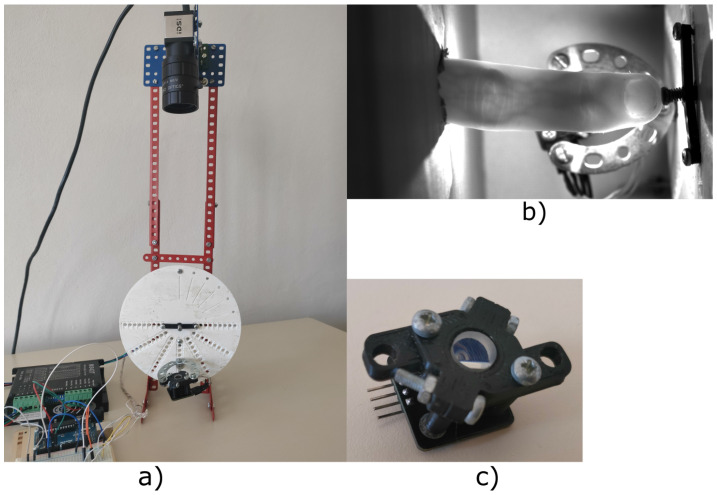
Test device (**a**) Image of the device without cover. (**b**) Image from the device with alignment light and finger. (**c**) Laser diode with powell lens to illuminate the finger.

Scanners used for recognition based of the bloodstream use illumination by light through finger (transmissive). The target of this testing device is to test the visibility of the bloodstream with different angles of illumination and the same intensity. This device was constructed at Brno University of Technology, Faculty of Information Technology as part of the grant named Reliable, Secure, and Efficient Computer Systems (FIT-S-20-6427). The device uses one light source for illumination of a finger, and this light source is rotated around the finger. A static camera captures an image of an illuminated finger from various angles. An image of the device shown in Figure 7.

The capture device consists of a camera (IDS Imaging UI-3360CP-NIR-GL R2) with a 25 mm lens (Edmund Optics, 25 mm, #86-572). The illumination part of the device is inspired by a multi-respective finger-vein scanner [2] and it is created from one NIR laser diode (808 nm) with a 70 degree powell lens. The illumination is placed on a stepper motor (Nema 23, 23HS22-2804S), which rotates with light around the finger. Using the stepper motor, we gain precise information about the angle of the light source. Arduino UNO is used to control the stepper motor driver (MT542T) and intensity of light generated by the laser diode. The construction of the device is made from Merkur kit. The whole device is covered by cardboard shading, which prevents interference of ambient light.

The scanning procedure consist of six steps, which will take multiple images of the finger with different positions of the light:

1. Setup start position —Place light source in front of camera; to diode the light, go directly to the camera. Place the finger between camera and light source, and align the position of the finger by touching the center pin. The start position is shown in Figure 7b).
2. Define light intensity—Change pulse width modulation (PWM) on Arduino. Final intensity of the light is defined by a histogram to prevent overexposure and visibility of the bloodstream at the start of scanning. The intensity of the light will not be changed from the end of this point to the end of the scanning procedure.
3. Start automatic scanning—The procedure starts by taking five images of the finger, with light going trough the finger.
4. Move counterclockwise—Shift the light by 10 degrees counterclockwise and take five images with the new position of the light. Repeat this step until light is moved by 130 degrees from the starting position. This maximal angle was limited by the camera field of view when the light source overlaps the finger.
5. Back to start position—Shift light back to the starting position and take five testing image for comparison. These testing images should be the same as the five starting images.
6. Move clockwise—Repeat step 4 in the clockwise direction and move the light to 130 degrees from the start position. After reaching this angle, move back to the start position to take the last five images.

**Figure 8:**
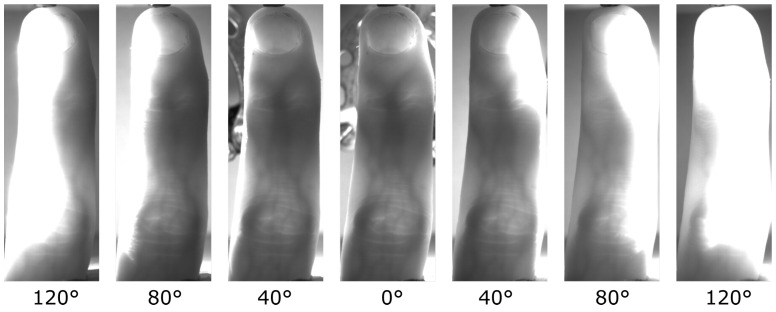
Multiple images of view to finger bloodstream with same intensity of light and different positions.

The result of the scan procedure is a dataset of 145 images. These images are pre-processed by removing a background with a rectangular image cutout of each image, because the finger is not moving and the position of finger is not changing. This part of the image is our region of interest (ROI). Cut-out images from one scanning cycle are shown in the Figure 8.

Using the procedure described in the previous part, we created a database. This database contains scans of 26 fingers, where 8 fingers were scanned from females, and the rest were from males. The database contains mostly middle and index fingers. Each scanned finger has a different intensity of light, based on the visibility of the bloodstream in the finger. The intensity of light was not changed during the scan to provide us simulation of one unchanged light source.

**Figure 9:**
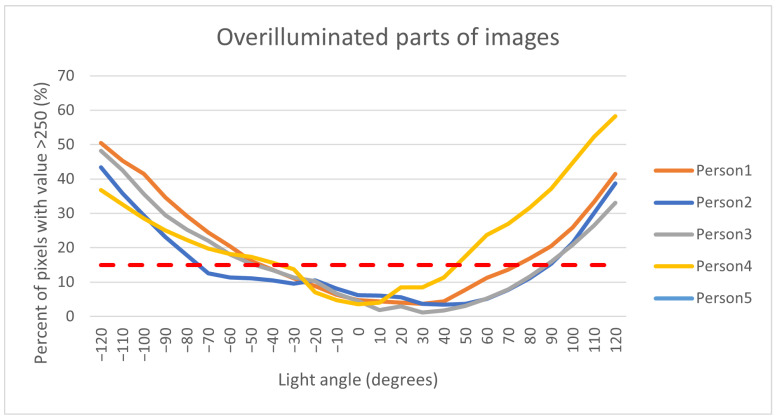
Display in the graph.

The first step of pre-processing data from the database is the extraction of the region of interest, which was cut from the captured image as the smallest possible rectangle. The second part of the pre-processing is the separate images by persons and position of the light source. All these images are in 8-bit grayscale format. The main part of processing data is based on the histogram. The overexposed part of the image is visible in the histogram as the highest value of the pixel, where we take value 250 of pixel as the border to define the overexposure pixel. Five images were taken for every position of the light. For each image, we compute the size of the overexposure part. From these five images, we compute average value as a percentage of overexposure for each finger and light position, and after that, from one light position we compute the median value of overexposure of all fingers for each person. These values are in Figure 9.

The results in Figure 9 show the rising overexposure part of the image with a decreasing angle between the camera and light source. The angle of 0 degrees in the graph defines the position of light in front of the camera. This position is also the biggest angle between the camera and the light. With increasing the angle from start position, light is reflected from the finger, which causes overexposure. The red line in the graph shows one of the possible borders between a good-quality image and an overexposed image. The result shows us the possibility of a visible finger bloodstream from multiple angles with a single light source almost to a 90 -degree angle. The information obtained from this device will be used for the design of a final proposed device.

### 4.2. Proposed Capturing Device Design

**Figure 10:**
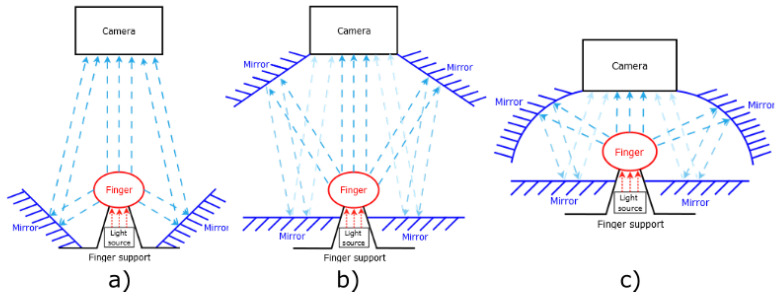
Three possible setups of the scanner (**a**) reflecting area as close as possible to finger; (**b**) reflection from the top of the finger; (**c**) curved reflection surface.

Our proposed device should use one light source position for illumination through the finger, as was proposed and realized on the testing device. The main part of the device is reflective surfaces, such as, for example, mirrors. These mirrors will give us a look from multiple angles in one finger. Possible positions of these reflecting surfaces are shown in Figure 10. The position of the finger is between the light and camera, where the finger is close to the light. The position of the finger has the best results on the testing device.

The first of the proposed devices in Figure 10a is using two mirrors as a reflective surface. These mirrors are based as close as possible to the finger. The size of the angle between the light and mirrors is lower then 75° in the comparison with the testing device, with the position of the camera greater then 105°. This type of device will have issue with overexposure finger on the mirrors, which show us the results of the testing device.

The second possibility is designed to remove the issue of the small angle between the light and mirrors from the first proposed device. To achieve this goal, it is necessary to move these mirrors to the space between the camera and finger, but the mirrors cannot cover the finger. To obtain the reflection image from these mirrors, it is necessary to use the next two mirrors because of the angle between mirrors and camera. The two additional mirrors will be positioned a little bit under the finger so as to not interfere with the direct view of the camera to the finger or reflection. This proposed device was constructed at Brno University of Technology, Faculty of Information Technology. The final version of the device was composed of:

- NIR sensitive camera Basler acA4112-30uc with lens 25 mm/F1.8 from Edmund Optics (type: 86572)
- Laser diode (LCU80B051A) with a light spectrum between 805 nm to 811 nm
- Powell lens with 75° fan angle from Edmund Optics
- Arduino Uno and circuit with LED Lighting Driver to control the intensity of light
- 4× Glass mirrors of 4 mm thickness used as reflecting surface
- Construction from the Merkur kit
- Plastic parts printed on a 3D printer for fixation position of the finger and as holder of mirrors

**Figure 11:**
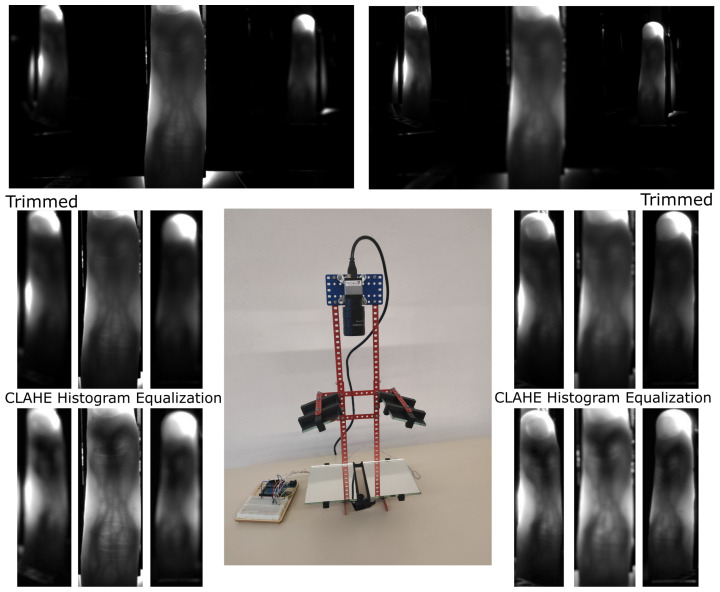
Center picture is image of constructed scanning device. Top left image is scan result focused on direct view on the finger. Top right image is scan focused on reflection of the finger. Second line of images is cut region of interest. Last line is result after using CLAHE histogram equalization.

The device is shown in the center in Figure 11. Two captured images of one finger are visible on the top, where the left side is focused on a direct view, and the right side is focused on a reflection of the finger. We cut the direct and reflected view on the finger, and this is visible in the second line of the image. The last line of images is after using the CLAHE histogram equalization algorithm to increase the visibility of finger veins.

From the resulting image in Figure 11, the needed two focusing depths are visible. One focus depth is for reflection from mirrors and second focus depth is for direct view on the finger. With the increasing number of reflection views, it will give rise to a number of focus depths. This issue might be solved by using a mechanical lens with turnable focus or a liquid lens. The number of images will be increasing with the number of focusing distances. It is not possible to create a single image with all reflections in focus. The second of the disadvantages is the various sizes of fingers in the image. However, this issue can be solved by post-processing.

The most recent version of the proposed device uses a curved mirror. The curved mirrors should give us multiple angles of view on one reflecting surface. Multiple angles of view give us more information to create a 3D model of a bloodstream. Curved mirrors deform the resulting image. These deformations can be compensated by post-processing. This can create missing data for reconstruction, which lead us back to the previously proposed device.

### 4.3. Advantages of the Proposed Device

The proposed capturing device is constructed from three basic parts, where two of these parts can be the same as parts in compared devices with some differences. The device from the University of Twente [13] uses three cameras with three light sources. The proposed device only uses one camera and one light source. Compared to the rotational devices, e.g., [2], the proposed capturing device eliminates the need for rotating parts, thus greatly decreasing the capturing time and mechanical complexity. Furthermore, the proposed device significantly reduces the acquisition time compared to the rotational devices and even compared to, e.g., the device proposed by the University of Twente [13] (as it captures the image with each camera sequentially).

The major advantages of the proposed device are the reduced production costs compared to the aforementioned multi-camera devices. As mentioned before, the production costs increase with the number of cameras and illumination modules, where our proposed devices only need one camera and illumination module in combination with mirrors and a few other parts (housing etc.).

To enable a cost comparison with previous devices by other authors (where the part costs are not available as they were not stated in their respective papers), we normalise the costs of the devices by the employed camera we used in a previous rotational capturing device [2], as well as our three-finger capturing device [38] as a reference. This camera is an IDS Imaging UI-ML-1240NIR near-infrared enhanced industrial camera with a 12 mm lens and costs roughly EUR 600. This normalisation is natural as the near-infrared camera usually contributes the biggest share of the total device costs. For the rotational capturing device [2], there are also mechanical parts for the rotation and the metal frame, which are about EUR 350, leading to total costs of roughly EUR 950 (including the control PCB, stepper motor, gear, illumination module, etc.). Our previous three-finger capturing device [38] only captures one view and does not involve rotating parts, but is based on the same industrial camera plus the illumination units, the control PCB, and the housing, which adds up to a total parts cost of roughly EUR 800 and EUR 900 for the LED and the laser based version, respectively. A device with three cameras and illumination units instead of one based on the IDS industrial camera to normalise the costs would result in total costs of at least EUR 2000. In addition, for our proposed device, the main share of the costs is the industrial camera. The next part of the device is the powell lens, whose price is around EUR 200. We calculated the total price of the testing device to be approximately EUR 900, which is less than two of the previous devices.

## 5. Conclusions

Three-dimensional or multi-perspective acquisition devices are one way to overcome the current problems in finger vein recognition, especially related to finger misplacement and longitudinal finger rotation. In the first part of this work, after giving a short introduction and medical background on finger vein recognition, we identified the potential problems and shortcomings with the current state-of-the-art, multi-perspective finger vein acquisition device designs, mainly either the long acquisition times (for rotational devices) or their complexity and thus, the high production costs.

In order to overcome these problems, we derived a novel multi-view finger vein acquisition device design in the second part of this work. In contrast to existing designs, which either involve rotating parts or multiple cameras and illumination modules, our proposed design is based on a single, fixed camera and a single illumination module in combination with several mirrors to be able to capture the finger at different rotational angles. Hence, it is able to capture all the multiple views in a single shot, decreasing potential finger movements during the acquisition (main problem with rotational devices). Due to the reduced number of cameras and illumination modules as compared to the existing designs, this novel design helps to reduce the complexity and costs of the multi-view finger vein acquisition device compared to the multi-camera devices. We tested several possible configurations to derive the optimal one in terms of sample image quality, and constructed a first prototype of the device and acquired several test finger vein images. The visual inspection of those samples looks quite promising and confirms the good sample image quality.

Our future work will include improvements to the capturing device itself to optimize the image quality, increase the number of mirrors to obtain more rotational angles, and reduce the distortions introduced by the mirrors. Once the sample quality is satisfying, we plan to acquire a full dataset in order to conduct finger vein quality as well as recognition performance evaluations based on these samples to demonstrate that our proposed device is able to achieve a reasonable recognition performance for practical applications. A particular application for the proposed device would be an access control system for a building in a high-security environment where the recognition performance of a single view capturing device is not sufficient. Another advantage of the multi-perspective finger vein acquisition devices is their increased resistance to presentation attacks, making it a suitable choice for a high-security environment. Thanks to the reduced price of our proposed device, it can be employed in large numbers to equip all entrance doors in such a building and will thereby greatly reduce the costs as compared to equipping one of the previously existing devices.

Further improvements of the acquisition device in future work will also include changing the position of the mirrors to remove multiple focuses of the camera, which will reduce the time needed to acquire a sample. This will reduce the number of captured images in one scan to reduce problems due to involuntary finger movements. The reduction in the number of images in one scan also enables the possibility of multiple scanning with different light sensitivities or wavelengths. With the fully functional version of the scanner device, we assume to create a database, where we will focus on a person with different levels of melanin, age, finger and sex to obtain base information on quality finger veins. The first post-process step will be implemented and will test existing algorithms for finger vein detection. This step will provide us with data to compare the quality with another existing device. The next step will be to implement and test some algorithms for the recognition, where algorithms will have two recognition possibilities. The first will compare two 3D models, and the second algorithm will use a 3D model and compare it to a captured 2D image. The second option should allow us to use an existing 2D scanning device for the recognition to eliminate the problem of finger rotation.

## Data Availability

Not applicable.
